# Moderators and short and long-term effects of a behavioral intervention on sedentary behavior among adults with depressive symptoms: results from a randomized clinical trial

**DOI:** 10.1007/s10865-026-00636-8

**Published:** 2026-04-17

**Authors:** Cecília Bertuol, Gregore Iven Mielke, Deborah Kazimoto Alves, Anne Ribeiro Streb, Marcelo Baggio do Amaral, Marcus Vinicius Veber Lopes, Kelly Samara da Silva, Giovani Firpo Del Duca

**Affiliations:** 1 Physical Education Department, Federal University of Santa Catarina, Florianópolis, 88040-900 Brazil; 2 School of Public Health, University of Queensland, Herston, 4006 Australia; 3 Postgraduate Program in Public Health, Federal University of Ceará, Fortaleza, 60714-90 Brazil; 4 CHEO Research Institute, Ottawa, ON K1H 8L1 Canada

**Keywords:** Sedentary behavior, Television, Computers, Cell phone use, Adults, Depressive symptoms

## Abstract

Although physical activity is recognized as an effective strategy for treating depressive symptoms, little is known about its impact on sedentary behavior (SB) when implemented in intervention settings. This study aimed to evaluate the short and long-term effects of a behavioral intervention on different indicators of SB and to identify potential moderators in adults with depressive symptoms. A randomized, controlled, and assessor-blinded clinical trial was conducted with adults exhibiting depressive symptoms. The 16-week intervention included two sessions per week and was designed based on principles of Self-Determination Theory. Theoretical and practical activities were offered with the main objective of increasing participants’ awareness of their lifestyle, especially related to 24-hour movement behaviors. SB was assessed through both accelerometry and validated self-report questionnaires. Generalized estimating equations were used to assess the effects of the intervention. Potential moderators (sex, age, and marital status) of the intervention’s effects were also analyzed. A total of 78 individuals (76.9% women, mean age 37.5 ± 10.2) were equally randomized into control (CG) and intervention (IG) groups. Overall, there was no significant effect on reducing SB. After the intervention, the CG showed an increase in leisure-time SB (*p* = 0.028) and in mentally passive SB (*p* = 0.018). Sex moderated the effects for cell phone use (*p* = 0.087), leisure-time SB (*p* = 0.042), and mentally passive SB (*p* = 0.050), with greater increases observed among men. Age also moderated leisure-time SB (*p* = 0.093), indicating that middle-aged adults spent less time in SB compared to younger adults. Marital status moderated SB measured by accelerometry (*p* = 0.078), with an increase in weekly SB hours among individuals with a partner. The intervention was not effective in reducing SB, either in the short or long term. Nonetheless, sex, age, and marital status emerged as important factors to consider in the design and evaluation of behavior change strategies.

*Trial registration* Brazilian Registry of Clinical Trials (ReBEC) RBR7466htj, April, 17 2023. Retrospectively registered.

**Supplementary Information:**

The online version contains supplementary material available at 10.1007/s10865-026-00636-8.

## Background

Technological advances in recent decades have led to significant changes in lifestyle, particularly an increase in time spent in sedentary behavior (Silva et al., 2021; Woessner et al., 2021). Activities such as watching television, using electronic devices (computers, tablets, cell phones), and remaining seated during transportation (e.g., driving or riding in vehicles) have become predominant across various daily contexts, including work, study, leisure, and commuting (Biddle et al., 2017; Gibbs et al., 2021; Silva et al., 2021). As a result of this widespread prevalence, sedentary behavior has been recognized as a risk factor for several non-communicable chronic diseases (Ekelund et al., 2019, 2020; Patterson et al., 2018; Wu et al., 2023), including mental health disorders such as depression (Gibbs et al., 2021; Huang et al., 2020; Zhai et al., 2015).

Depressive symptoms, in turn, have also gained prominence due to their high prevalence, functional impact, and contribution to the global burden of disease (GBD 2019 Mental Disorders Collaborators, 2022; Thornicroft & Semrau, 2019). However, the relationship between sedentary behavior and depression appears to be bidirectional (Hiles et al., 2017): while prolonged sedentary time is associated with an increased risk of depressive symptoms, individuals with more severe symptoms tend to spend longer periods engaged in sedentary activities (Schuch et al., 2017; Vancampfort et al., 2017).

In this context, interventions aimed at improving depressive symptoms may yield additional benefits by reducing sedentary behavior. Lifestyle-based strategies, particularly those focused on promoting physical activity, have emerged as promising alternatives for managing depression (Heissel et al., 2023; Pearce et al., 2022; Recchia et al., 2022; Singh et al., 2023). Evidence suggests that physically active individuals tend to spend less time in sedentary behavior (Bull et al., 2020) and that such interventions can contribute to a healthier and more active lifestyle. For example, a study conducted with individuals with serious mental illness reported a simultaneous reduction in sedentary behavior and an increase in physical activity levels following an intervention that included an initial education session, fortnightly coaching, pedometer provision, and access to a weekly walking group (Williams et al., 2019).

Despite these advances, clinical trials specifically targeting sedentary behavior in adults with depressive symptoms remain scarce, particularly in non-occupational settings (Murtagh et al., 2020). Moreover, based on the existing evidence, several studies have reported weak, null, or mixed associations, possibly due to differences in study design, measurement methods, and sample characteristics (Kandola et al., 2021; Werneck et al., 2023). These inconsistencies highlight the complexity of this relationship and the importance of further research in populations with clinically relevant depressive symptoms. Furthermore, most studies examining movement behaviors in individuals with depressive symptoms have focused on short-term effects (Ashdown-Franks et al., 2018). Although some findings suggest positive outcomes up to six months post-intervention (Williams et al., 2019), there remains limited evidence regarding the long-term maintenance of these effects, particularly with respect to sedentary behavior (Ashdown-Franks et al., 2018). The absence of follow-up assessments hinders our understanding of the sustained effectiveness of such strategies and highlights the need for studies including medium and long-term evaluations.

Another aspect that remains underexplored is the identification of potential moderators of intervention effects. Sociodemographic variables such as sex, age, and marital status may influence sedentary behavior patterns and the degree to which individuals respond to physical activity promotion strategies (Ferrara et al., 2022; O’Donoghue et al., 2016 ; Zhang et al., 2022). Beyond their associations with sedentary behavior and depressive symptoms (Schuch et al., 2017; Ferrara et al., 2022; O’Donoghue et al.,^2016^), these characteristics may also reflect differences in self-care orientations and social support availability – factors that can shape engagement with behavioral interventions. For instance, evidence suggests that women and older adults tend to adopt a broader range of self-care and health-promoting behaviors compared to their counterparts (Deeks et al., 2009; Gorini et al., 2025), which supports the hypothesis that these groups might react differently to structured behavioral interventions. In addition, relational factors such as marital status may impact lifestyle and the level of social support available for behavior change, particularly in emotionally vulnerable contexts (Thomas et al., 2022; Huang et al., 2024). Considering these moderators can enhance our understanding of for whom, and under what conditions, interventions are most effective.

Taken together, the following arguments support the undertaking of this study: (1) few studies have simultaneously examined short and long-term effects of behavioral interventions on sedentary behavior among adults with clinically relevant depressive symptoms, particularly within a randomized controlled trial design. In the review by Ashdown-Franks et al. (2018), among 32 included studies, only five reported sedentary behavior outcomes – none as a primary outcome – and only one showed a significant reduction in sitting time after the intervention. Importantly, no study assessed long-term effects, and the studies that did report sedentary behavior outcomes were uncontrolled trials; and (2) the inclusion of sociodemographic moderators also offers a novel contribution to understanding differential responses to such interventions.

Given the above, this study aimed to evaluate the short-term (post-intervention) and long-term (six-month follow-up) effects of a behavioral intervention on different indicators of sedentary behavior and to identify potential moderators (sex, age, and marital status) among adults with depressive symptoms.

## Materials and methods

### Study design

This study, also known as *Vincular* Project, is a randomized, controlled, assessor-blinded clinical trial with two parallel groups (control and intervention) conducted following the CONSORT guidelines (Additional file 1). The research protocol adheres to the Declaration of Helsinki, was approved by the Human Research Ethics Committee (CAAE: 60378122.1.0000.012), and all participants provided written informed consent before enrollment. The study protocol was registered in the Brazilian Clinical Trials Registry (ReBEC) under the code RBR-7466htj. Further methodological details can be found in it (Bertuol et al., 2024).

### Participants

The sample of the present study was composed of adults with depressive symptoms. The main eligibility criterion was presenting moderate to severe depressive symptoms, based on a score ≥ 9 on the Patient Health Questionnaire (PHQ-9) (Santos et al., 2013). Additional criteria included residing in Florianópolis and metropolitan cities, being between 20 and 59 years old, owning a smartphone, a tablet or other electronic device, having internet access, being available during scheduled meeting times, and not being pregnant or in the postpartum period. Participants were excluded if they required specialized psychiatric treatment for severe mental illnesses (e.g., schizophrenia), based on self-reported diagnosis; if they were at risk of suicide (indicating “several days,” “more than half the days,” or “nearly every day” for the suicide risk question on the PHQ-9); or if they had physical illnesses or limitations that prevented participation in the study. It is noteworthy that psychoeducational materials on mental health were provided, and referrals were made for individuals who were not included in the study and those identified as being at risk of suicide (Bertuol et al., 2024).

### Care providers

Three exercise specialists with expertise in health-related physical activity were responsible for standardizing the intervention procedures. Additionally, external guests with training in various health-related fields contributed to specific activities and sessions. None of these individuals were involved in participant evaluations. Given the specific nature of the intervention groups, blinding was not feasible for participants or care providers (Bertuol et al., 2024).

### Recruitment, screening, and selection

Participants were recruited through social media campaigns, the distribution of letters and pamphlets, and advertisements in local newspapers and podcasts between November 2022 and January 2023. Interested individuals signed an informed consent form and completed an initial screening questionnaire, both conducted online. Those who did not meet the eligibility criteria were excluded from the study. While direct referrals to professionals were not made, participants were advised to seek appropriate care, and a list of options was provided. Eligible participants were invited to an orientation meeting for further clarification, completion of additional assessments, and distribution of accelerometers (Bertuol et al., 2024).

### Sample size, randomization, and blinding

The sample size was calculated using G*Power software (version 3.1.9.2), based on the primary outcome of the larger *Vincular* Project (depressive symptoms) assessed at three time points (baseline, post-intervention, and follow-up). The analysis followed an ANOVA model for repeated measures with two groups and three measurements (within-between interaction), considering a medium effect size of 0.25 (DeVellis, & Thorpe, 2021), a statistical power of 80%, a significance level of 5% for two-tailed tests, a correlation of 0.5 between repeated measures, and a correction for non-sphericity set to 1, based on default recommendations (Cohen, 2013). According to Cohen (2013), f = 0.10 represents a small effect, f = 0.25 a medium effect, and f = 0.40 a large effect, indicating the strength of the relationship between variables. This calculation indicated the need for 28 participants. To account for a possible dropout rate of up to 50%, the sample size was doubled, resulting in a final total of 56 participants, who were randomly assigned to either the control group (CG) or the intervention group (IG) (Bertuol et al., 2024).

Although the original sample size calculation for the Vincular Project was based on depressive symptoms, we performed an additional power analysis specifically for the sedentary behavior outcomes. The analysis followed the method proposed by Liu and Liang (1997) for studies with correlated observations and was conducted in R using the longpower package (version 1.0.27). For a design with three repeated measurements modeled through Generalized Estimating Equations (GEE) (exchangeable correlation structure), we assumed: power = 0.80, α = 0.05, standard deviation = 100 min/day for sedentary time, and a conservative pre–post correlation of 0.50. Under these assumptions, the required sample size to detect a between-group difference in change of 30 min/day was 262 participants, whereas detecting a difference of 60 min/day required 66 participants. Our analytic sample of 78 participants was therefore insufficient to detect modest SB changes but adequate for large effects.

Randomization was performed using the online tool randomizar.org, with stratification based on sex (male and female), age (in years), and depressive symptoms (PHQ-9 scores). The allocation sequence was generated by a researcher not directly involved in the study, and the allocation list was kept concealed from the research staff (undergraduate and graduate students who were previously trained and were not involved in other aspects of the study) throughout the trial to ensure their blinding (Bertuol et al., 2024). However, due to the behavioral nature of the intervention, participant blinding was not feasible, which may represent a potential source of performance bias inherent to this type of study.

### Experimental procedure

The program lasted 16 weeks, with two weekly sessions held on alternate days, totaling 32 sessions. Each session lasted approximately 90 min and was conducted primarily in person (80%). Both practical and theoretical activities were planned with the aim of increasing participants’ awareness of their living conditions and health, by exploring 24-hour movement behaviors (physical activity, sedentary behavior, and sleep), with a particular emphasis on physical activity, and their relationship with depressive symptoms. The program was grounded in the principles of Self-Determination Theory (Deci & Ryan, 1985, 2000), focusing on the promotion of self-determined motivation and the sustainable adoption of healthy behaviors. To this end, techniques supporting basic psychological needs (autonomy, competence, and relatedness), as described by Teixeira et al. (2020), were employed to foster behavior change and improve physical and mental health. All sessions were conducted by health professionals who were aware of the participants’ mental health conditions and applied strategies to create a safe and inclusive environment that allowed each participant to engage at their own pace and comfort level. The activities were not simplified or clinically adapted but were delivered in a humanized and supportive manner consistent with Self-Determination Theory principles (Bertuol et al., 2024). Gamification strategies were also incorporated to enhance participant engagement throughout the sessions.

**Fig. 1:**
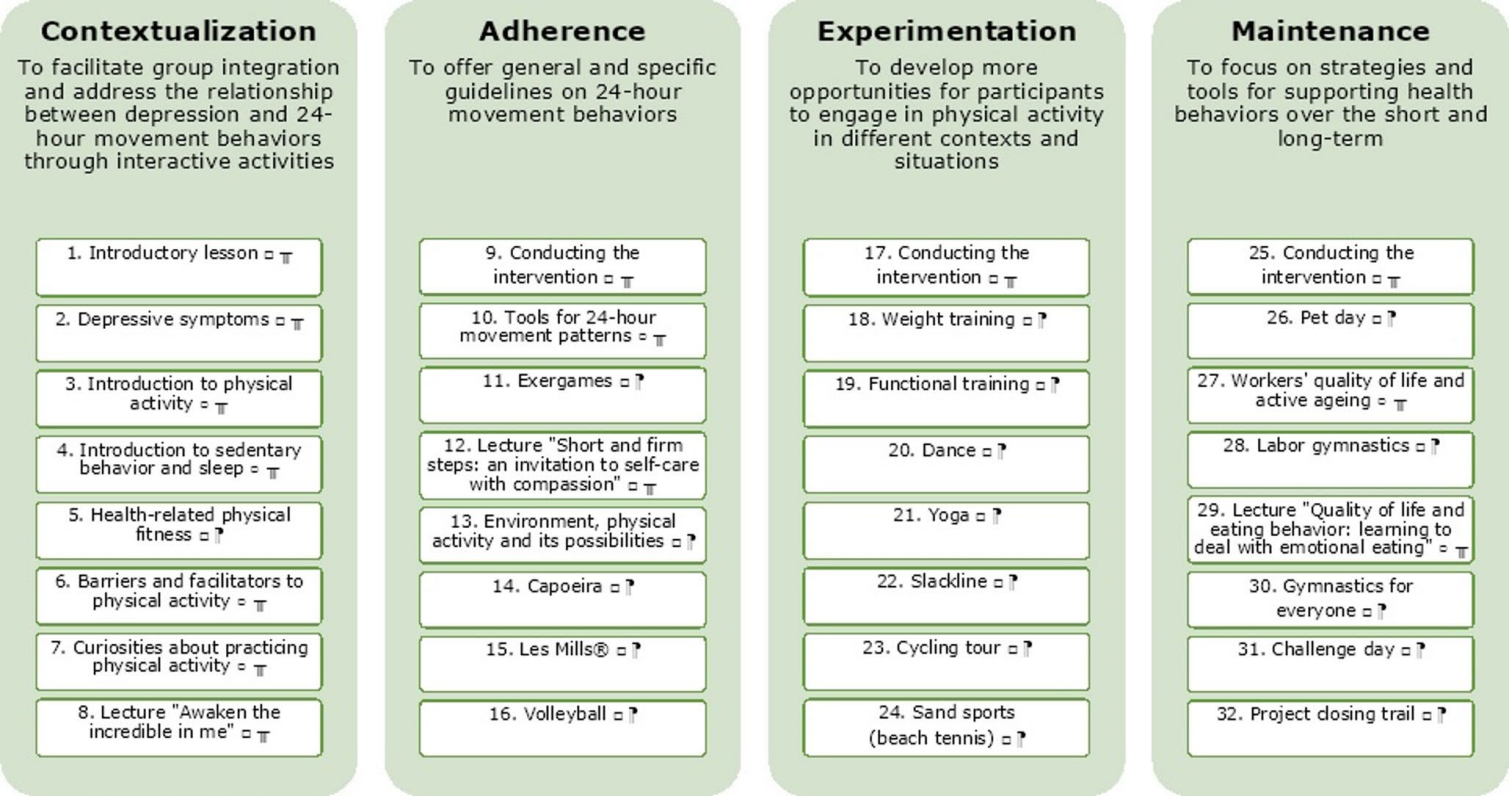
Organizational structure of the *Vincular* project. Notes: □ = face-to-face meeting; ○ = online meeting; ╥ = theoretical/educational sessions; ⁋ = practical sessions

The sessions were organized into four blocks of eight meetings: (1) contextualization (group integration and reflections on depressive symptoms and movement behaviors); (2) adherence (general and specific practice guidelines); (3) experimentation (experiencing multiple contexts and modalities); and (4) maintenance (long-term strategies), as illustrated in Fig. 1. Activities included lectures, group discussions, distribution of educational materials, and practical sessions (e.g., yoga, capoeira, exergames, cycling, animal-assisted walking, resistance training, functional training, and group fitness classes), as well as the involvement of family and friends. It is worth noting that eight sessions (4, 8, 10, 11, 17, 25, 27, and 28 in Fig. 1) focused more extensively on the topic of sedentary behavior, addressing its definition, harmful health effects, and practical suggestions for reducing it in daily life. A WhatsApp group was also created to provide continuous support (e.g., answering questions, sharing materials, and recording tasks).

### Variables and instruments

The CG maintained their usual routines but received biweekly messages containing psychoeducational material on mental health, physical activity recommendations, sedentary behavior reduction, and sleep improvement. It is important to note that this group also served as a waitlist control, with the expectation of receiving the full intervention later if positive effects were observed on the primary or secondary outcomes. Further details of the experimental procedures can be found in the study protocol (Bertuol et al., 2024).

## Variables and instruments

Assessments were conducted at three time points: (1) baseline (January 2023); (2) post-intervention, immediately after the intervention (May 2023); and (3) follow-up, six months post-intervention (November 2023).

Sedentary behavior was assessed through both self-reported and objectively measured using accelerometers. The Simple Physical Activity Questionnaire (SIMPAQ) was employed to provide a validated measure for individuals living with mental illnesses. The SIMPAQ evaluates physical activity and sedentary behavior by capturing time spent in the past seven days in the following contexts: (a) in bed; (b) sedentary behavior; (c) walking for exercise, recreation, or commuting; (d) exercising; and (e) other activities, such as work-related tasks or household chores (Rosenbaum et al., 2020). For this study, only sedentary behavior was analyzed. Additionally, sedentary behavior was measured using the questionnaire proposed by Mielke et al. (2020). This instrument assesses the time spent in sedentary behavior on a regular weekday and considers different indicators, such as: (1) watching television, (2) using a computer, notebook or tablet at home, (3) using a cell phone to play games, access the internet or applications, (4) sitting at work, (5) sitting at school, technical course, college or other course, and (6) sitting while traveling by car, motorcycle or bus.

Furthermore, for this study, different sedentary behavior indicators were grouped based on the context in which they occurred. One classification considered the domain of the behavior: (1) leisure: watching television, using a computer, notebook or tablet at home, and using a cell phone to play games, access the internet, or use applications; (2) occupational: sitting while at work, school, technical courses, college, or other educational activities; and (3) passive commuting: sitting while traveling by car, motorcycle, or bus. Another classification was based on the level of cognitive effort/engagement involved (Hallgren et al., 2018; Huang et al., 2020; Werneck et al., 2023), and included two categories: (1) mentally-passive: watching television, using a computer, notebook or tablet at home, using a cell phone to play games or access the internet/applications, and sitting during passive commuting; and (2) mentally-active: sitting while working or attending school, technical courses, college, or other educational activities. However, alternative categorizations were created and analyzed (Additional file 2), as the evaluators did not have access to the content participants accessed on their cell phones and computers, which allowed for different interpretations. Table 1 provides information on the assessment methods of sedentary behavior, the analyses conducted, as well as the classifications, combinations, and treatment approaches used.

**Table 1:** Methods for assessing sedentary behavior, analyses conducted, and corresponding classifications, combinations, and treatment approaches

Variables evaluated	Analyses	Classifications	Combinations	Treatment approaches of the variables
SB by accelerometer	Main analyses	Total SB duration		SB by accelerometer
SB by SIMPAQ				SB by SIMPAQ
Watching television Using a cell phone to play games, access the internet or applications Using a computer, notebook or tablet at home Sitting at work Sitting at school, technical course, college or other course Sitting while traveling by car, motorcycle or bus		Types of SB		TV time
				Cell phone use
				Computer at home
				Sitting time (work and school) ○
				Sitting time (commuting) □
		Domains of SB	Combination 1	Leisure (TV + cell phone + computer)
				Occupational (sitting time at work and school) ○
				Passive commuting (sitting while traveling by car, motorcycle or bus) □
		Levels of cognitive effort of SB	Combination 1	Mentally-passive (TV + commuting + cell phone + computer)
				Mentally-active (sitting time at work and school) ○
	Alternative categorizations (Additional file 2)	Domains of SB	Combination 2	Leisure (TV + cell phone)
				Occupational (sitting time at work and school + computer) ∂
				Passive commuting (sitting while traveling by car, motorcycle or bus) □
		Levels of cognitive effort of SB	Combination 2	Mentally-passive (TV + commuting)
				Mentally-active (sitting time and work and school + cell phone + computer)
			Combination 3	Mentally-passive (TV + commuting + cell phone)
				Mentally-active (sitting time at work and school + computer) ∂
			Combination 4	Mentally-passive (TV + commuting + computer)
				Mentally-active (sitting time at work and school + cell phone)

SB, sedentary behavior; SIMPAQ, Simple physical activity questionnaire; ○, □, ∂ = similar combinations

Objectively assessed sedentary behavior was obtained using ActiGraph GT3X + and wGT3X + accelerometers (ActiGraph Corporation, Pensacola, Florida, USA) worn on the non-dominant wrist for seven consecutive days. Participants adhered to a 24-hour usage protocol, removing the device only for activities involving water submersion. Acceleration signals were collected at 30 Hz, then underwent a self-calibration process and were converted to the ENMO (Euclidean Norm Minus One) metric, expressed in milligravitational units (mg) (Migueles et al., 2019). The data were analyzed in 5-second epochs, with sedentary behavior defined as activities < 35.6 mg (Hildebrand et al., 2014). Wear time was considered valid if participants used the accelerometer for at least three days (16 h per day), including two weekdays and one weekend day. A “measurement day” was defined as the time between waking periods, which could exceed or fall short of 24 h. Accelerometer data were programmed and downloaded using Actilife software (version 6.8.11 for Windows), and raw data were analyzed with the GGIR package (version 2.9.5).

### Safety procedures and adverse events

Given the nature of the intervention and the clinical condition of the sample, safety procedures were put in place to protect participants. First, the research team’s contact information (telephone and email) was provided in the informed consent form and all study materials. Participants were encouraged to contact the team with any concerns or questions during the intervention. All messages and emails were responded to within 24 h, and face-to-face conversations were also conducted to provide individualized support and ensure participant safety. Additionally, if a participant missed two consecutive sessions, the team reached out to check on their well-being. In cases of withdrawal, the reasons for discontinuation were explored and documented.

### Statistical analysis

**Fig. 2:**
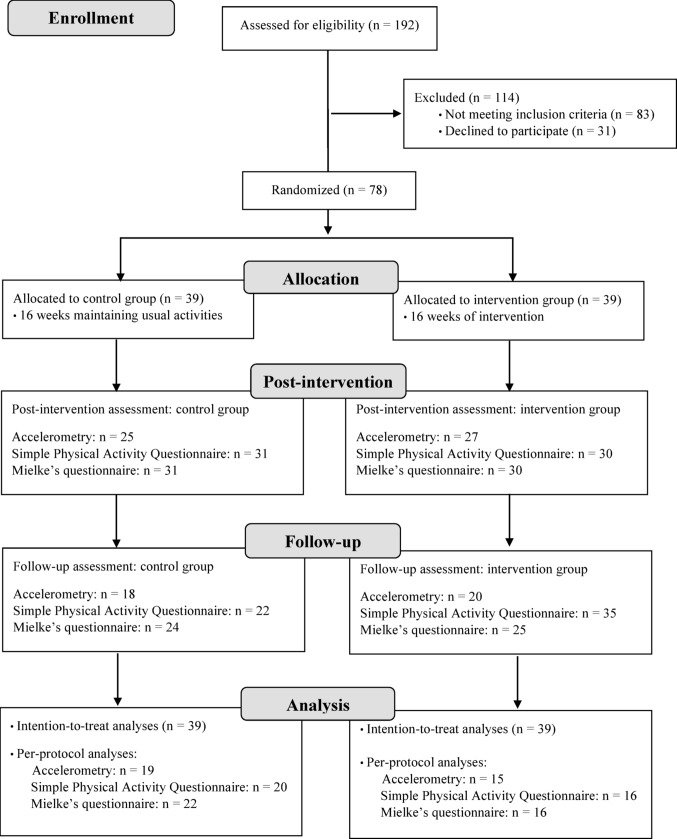
Flow diagram from *Vincular* project

To evaluate the short and long-term effects of the intervention on sedentary behavior, both per-protocol and intention-to-treat analyses were conducted. In the per-protocol analysis (Additional file 2), only participants who attended the intervention activities until the final session, complied with the protocol (with at least 50% overall attendance and in each of the four intervention blocks), and were properly assessed at all three time points (baseline, post-intervention, and follow-up) were included. In the intention-to-treat analysis, all participants who were randomized and participated in the three data collections were considered, regardless of their adherence to the intervention, any protocol deviations, or other issues that may have occurred after randomization. Missing data from the post-intervention and follow-up assessments were imputed using multiple imputation via the fully conditional specification (FCS) method, which is the default approach in SPSS. Predictive mean matching (PMM) was used for continuous variables, with five imputed datasets generated and pooled according to Rubin’s rules. The imputation model included depressive symptoms, sex, age, and time-varying physical activity indicators, sedentary behavior, and sleep duration and quality as predictors. To account for the interaction effects between repeated measurements (i.e., baseline, post-intervention, and follow-up) and conditions (i.e., control and intervention), imputations were performed separately for each condition. The number of missing cases at each assessment point is shown in the study flow diagram (Fig. 2).

For comparisons between periods and groups, GEE were fitted with a normal distribution and identity link. An independent working correlation structure was specified with robust (sandwich) standard errors, and type-III Wald tests were used. Pairwise post-hoc comparisons of estimated marginal means for group, time, and group*time were adjusted using Bonferroni correction. The overall significance level was set at *p* ≤ 0.05. Exploratory moderation analyses were conducted to examine whether the intervention effects varied according to participant characteristics. Three-way interaction terms (group*time*moderator) were tested, considering sex (men and women), age (20 to 36 years and 37 to 59 years, based on the sample’s mean and median values), and marital status (with a partner, defined as being married or in a stable union for more than six months vs. without a partner, which included individuals who were single, widowed, separated, or divorced) as moderators. Given the reduced statistical power and uneven subgroup sizes – particularly for sex – and following methodological recommendations (Montgomery, 2013), a *p* < 0.10 threshold was adopted as a screening criterion to identify potentially meaningful effects, while the conventional *p* ≤ 0.05 level was retained for all primary analyses. Simple main effects were examined for significant three-way interactions. Effect sizes were presented as partial eta squared ($\:{\eta\:}_{p}^{2}$), with the following interpretations: small (0.02 ≤ $\:{\eta\:}_{p}^{2}$ < 0.13), medium (0.13 ≤ $\:{\eta\:}_{p}^{2}$ < 0.26), and large ($\:{\eta\:}_{p}^{2}$ ≥ 0.26) (Lakens, 2013). All analyses were performed using the Statistical Package for the Social Sciences (SPSS, version 23.0).

## Results

A total of 78 individuals were randomized into the CG and IG (39 adults in each group). An additional 83 individuals were excluded for not meeting the eligibility criteria. The main reasons were disease or physical limitation (*n* = 14), pregnancy (*n* = 1), unavailability on the required days (*n* = 13), living outside the designated area (*n* = 8), suicidal ideation (*n* = 53), and receiving specialized treatment (*n* = 7). It is important to note that the number of reasons exceeds 83 because several individuals met more than one exclusion criterion. At the end of the intervention, 61 participants completed the questionnaires, and 52 adhered to the accelerometer protocol, providing valid data. At the follow-up assessment, the number of participants completing Mielke’s questionnaire, SIMPAQ, and accelerometer protocol decreased to 49, 47, and 38, respectively (Fig. 2). The dropout rate for the IG was 59%, with a total of 23 participants discontinuing the program due to difficulty balancing schedules (*n* = 7), personal issues (*n* = 7), work or study commitments (*n* = 4), distance (*n* = 3), or loss of interest (*n* = 2). No adverse events were reported or observed throughout the intervention. Furthermore, no significant differences were found between the CG and IG groups at baseline in terms of sociodemographic variables (sex, age, skin color, marital status, and education), clinical variables (depressive symptom score, mental illness diagnosis, depression alone, and medication use for depression), and related to physical activity (light, moderate, and vigorous physical activity by accelerometer, and walking for exercise, recreation, or commuting; exercising; and other activities by SIMPAQ), and sleep indicators (sleep duration by accelerometer, time in bed by SIMPAQ, and sleep quality). A detailed characterization of the sample is presented in Additional file 2.

The short and long-term effects of the intervention on sedentary behavior, as well as its potential moderators, analyzed under the intention-to-treat principle, are presented in Table 2. At the end of the intervention, no statistically significant effects were observed for the sedentary behavior outcomes investigated. The comparison between post-intervention and follow-up periods indicated a significant increase in leisure-time sedentary behavior (*p* = 0.028) and in mentally passive sedentary behavior (*p* = 0.018), but only in the CG. Sex moderated the effects on cell phone use (*p* = 0.087), leisure-time sedentary behavior (*p* = 0.042), and mentally passive sedentary behavior (*p* = 0.050). A similar pattern was observed across these three indicators, with the between-group difference in change (diff-in-diff) increasing among men and decreasing among women (Table 3). Leisure-time sedentary behavior was also moderated by age (*p* = 0.093): the diff-in-diff from baseline to post-intervention was − 2.3 h/week among middle-aged adults (aged 37–59 years) compared to 2.1 h/week among younger adults (aged 20–36 years); and − 3.3 h/week among middle-aged adults compared to − 0.6 h/week among younger adults from baseline to follow-up (Table 3). Marital status moderated the effect on sedentary behavior measured by accelerometry (*p* = 0.078), with an increase in hours per week among participants with a partner and a decrease among those without a partner, both from baseline to post-intervention and from baseline to follow-up (Table 3).

**Table 2:**
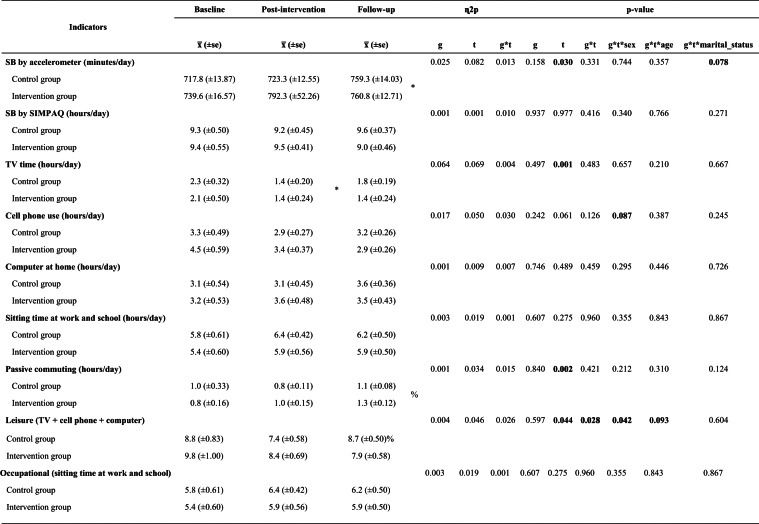

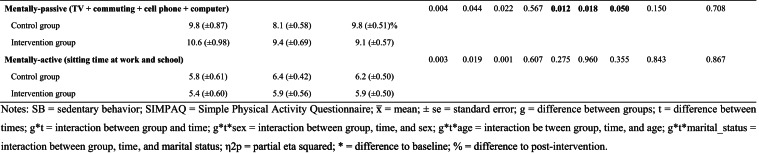
Short and long-term effects and moderators of a behavioral intervention on different indicators of sedentary behavior in adults with depressive symptoms (*n* = 78)

**Table 3:** Short and long-term effects of a behavioral intervention on different indicators of sedentary behavior according to sex, age, and marital status (*n* = 78)

Indicators	Men				Women			
	Baseline	Post-intervention	Follow-up	diff-in-diff (a / b)	Baseline	Post-intervention	Follow-up	Diff-in-diff (a / b)
	x̅ (± se)	x̅ (± se)	x̅ (± se)		x̅ (± se)	x̅ (± se)	x̅ (± se)	
*Cell phone use*				2.0 / 0.7				− 1.4 / − 2.1
Control group	4.9 (± 1.42)	3.2 (± 0.68)	3.9 (± 0.51)		2.9 (± 0.43)	2.8 (± 0.28)	3.0 (± 0.29)	
Intervention group	2.9 (± 0.61)	3.2 (± 0.75)	2.6 (± 0.38)		5.0 (± 0.72)	3.5 (± 0.42)	3.0 (± 0.31)	
*Leisure (TV + cell phone + computer)*				4.0 / 2.0				− 1.2 / − 3.0
Control group	11.9 (± 1.37)	8.2 (± 1.19)	9.4 (± 0.80)		7.8 (± 0.92)	7.1 (± 0.66)	8.4 (± 0.60)	
Intervention group	8.2 (± 1.48)	8.5 (± 1.25)	7.7 (± 1.32)		10.3 (± 1.20)	8.4 (± 0.81)	7.9 (± 0.63)	
*Mentally-passive (TV + commuting + cell phone + computer)*				5.3 / 2.7				− 0.9 / − 2.6
Control group	13.3 (± 1.90)	8.7 (± 1.10)	10.6 (± 0.84)		8.8 (± 0.90)	8.0 (± 0.67)	9.6 (± 0.60)	
Intervention group	8.8 (± 1.32)	9.5 (± 1.10)	8.8 (± 1.22)		11.1 (± 1.19)	9.4 (± 0.84)	9.3 (± 0.64)	

	20 to 36 years				37 to 59 years			
*Leisure (TV + cell phone + computer)*				2.1 / -0.6				− 2.3 / − 3.3
Control group	9.5 (± 1.32)	6.2 (± 0.071)	8.2 (± 0.80)		8.0 (± 0.94)	8.7 (± 0.83)	9.2 (± 0.57)	
Intervention group	10.8 (± 0.073)	9.6 (± 0.89)	8.9 (± 0.82)		8.8 (± 1.87)	7.2 (± 0.99)	6.7 (± 0.72)	

	With a partner				Without a partner			
*SB by accelerometer*				239.0 / 71.3				− 18.0 / − 52.1
Control group	726.3 (± 30.91)	740.6 (± 25.59)	764.2 (± 31.59)		714.0 (± 14.52)	715.6 (± 13.86)	757.0 (± 14.60)	
Intervention group	668.9 (± 39.32)	922.2 (± 192.43)	778.1 (± 22.62)		764.0 (± 15.25)	747.6 (± 16.32)	754.9 (± 15.05)	

SB = sedentary behavior; x̅ = mean; ± se = standard error; diff-in-diff a = [(post-intervention value from IG – baseline value from IG) – (post-intervention value from CG – baseline value from CG)]; diff-in-d iff b = [(follow-up value from IG – baseline value from IG) – (follow-up value from CG – baseline value from CG)]

The same comparisons described above were conducted per protocol and are presented in Additional File 2. Although no significant short- or long-term effects were found for sedentary behavior, sex and age moderated some specific indicators. Overall, women reduced their sedentary time, whereas men showed an increase. With respect to age, middle-aged adults reduced their sedentary time, while younger adults showed increased levels.

Exploratory analyses using alternative categorizations were also conducted and are presented in Additional File 2, for both intention-to-treat and per-protocol analyses. The findings were consistent across both approaches, with notable moderation by sex, particularly for indicators involving cell phone use. Once again, the data indicated a reduction in this behavior among women and an increase among men.

## Discussion

This study aimed to evaluate the short (post-intervention) and long-term (six-month follow-up) effects of a behavioral intervention on different indicators of sedentary behavior and to identify potential moderators (sex, age, and marital status) in adults with depressive symptoms. Overall, the intervention did not lead to significant reductions in sedentary behavior. However, significant group*time interactions were found for leisure sedentary behavior and mentally passive sedentary behavior, both of which increased in the CG from post-intervention to follow-up. Additionally, sociodemographic characteristics moderated the intervention’s effects. While age and marital status moderated only one sedentary behavior indicator each (leisure and accelerometer-based sedentary behavior, respectively), sex moderated three indicators (cell phone use, leisure sedentary behavior, and mentally-passive sedentary behavior).

Although the intervention was designed to target multiple movement behaviors, it did not yield satisfactory effects in reducing sedentary behavior. Alongside physical activity, sedentary behavior and sleep form the set of so-called 24-hour movement behaviors, a concept that promotes an integrated view of waking and resting periods throughout the day (Chaput et al., 2014; Tremblay et al., 2010). Within this framework, these behaviors can be interpreted in two distinct ways: (1) as interdependent, since increasing time spent in one behavior necessarily reduces time available for another; or (2) as independent, as it is possible, for instance, to meet physical activity guidelines while still accumulating prolonged periods of sedentary behavior during the day.

Despite growing interest in the relationship between sedentary behavior and depression, most available evidence is derived from observational studies, particularly cross-sectional and longitudinal designs. Experimental data on the mental health effects of reducing sedentary behavior remain scarce. Instead, many trials have focused on inducing sedentary behavior in otherwise active individuals to assess its impact on mood and depressive symptoms (Blough & Loprinzi, 2018; Edwards & Loprinzi, 2016; Endrighi et al., 2016). In one such study, participants were instructed to eliminate exercise and reduce daily steps for one week, resulting in significant short-term increases in depressive symptoms that returned to baseline after resuming normal activity (Edwards, & Loprinzi, 2016). More recently, a systematic review and meta-analysis found that lifestyle interventions, mostly multicomponent ones involving motivational counselling, self-monitoring, and digital tools, can reduce sedentary behavior in clinical populations (Niest et al., 2021). However, representation of individuals with mental health conditions, including depressive symptoms, was limited.

In this study, we focused exclusively on sedentary behavior, without considering physical activity or sleep, which limited a more comprehensive understanding of potential compensatory or interrelated effects. While this methodological choice allowed for a more detailed investigation of each behavior in separate analyses, it also restricts our ability to interpret why sedentary behavior remained unchanged. One explanation could be that changes in physical activity and/or sleep were also insufficient, thereby limiting their potential influence on sedentary behavior (Bull et al., 2020; Chaput et al., 2014; Segura-Jiménez et al., 2022; Tremblay et al., 2010). Another possibility is that, although sedentary behavior was addressed at various stages of the intervention (e.g., through conceptual content, practical guidance, and behavioral strategies) the primary emphasis was placed on promoting physical activity. As a result, participants may not have perceived sedentary behavior as an independent target with distinct health risks, but rather as something indirectly addressed through increased physical activity.

Certain domains of sedentary behavior, particularly those related to occupational contexts, also tend to be less modifiable (Bailey, 2021; Holmes et al., 2021; Pronk, 2021). Professionals who remain seated for extended periods at work may lack the autonomy or resources to alter this pattern. Strategies such as introducing brief, regular breaks or adopting sit–stand workstations have been proposed as feasible ways to mitigate the health risks of sedentary behavior and should be considered in future interventions or occupational health policies (Parés-Salomón et al., 2024). Moreover, although the present study was designed as an integrated behavioral program grounded in Self-Determination Theory, in which physical, educational, psychosocial, and gamification strategies act synergistically to support psychological need satisfaction, it is not possible to determine which specific strategies may have been more or less effective. Future studies could adopt factorial or dismantling designs to disentangle the relative contribution of individual components.

The absence of significant reductions in sedentary behavior may also be related to specific clinical and behavioral characteristics of the sample. More than half of the participants were under pharmacological treatment for depression and many had current or previous experiences with psychotherapy (Additional file 2), suggesting that they were already receiving ongoing care and might have limited responsiveness to additional behavioral strategies. Antidepressant use is known to influence daily energy levels, fatigue, and sleep–wake cycles, which can indirectly affect sedentary time and motivation to change lifestyle patterns (Kandola et al., 2019). Furthermore, although participants did not meet international physical activity recommendations, baseline values indicated that they were already sufficiently active for this population, with relatively high levels of light and moderate activity (Additional file 2). This profile reduces the potential for behavioral change, as sedentary time might play a compensatory role in managing low mood or conserving energy – a feature often observed in depressive symptomatology (GBD 2019 Mental Disorders Collaborators, 2022). The null or inconsistent results observed may also reflect limited statistical power due to sample attrition and potential measurement constraints, which could have reduced sensitivity to detect small or domain-specific changes in sedentary behavior. Therefore, reducing sedentary behavior in psychiatric populations may require more individualized and clinically integrated approaches that consider medication effects, motivational deficits, and the functional meaning of inactivity in daily life.

Another finding that deserves attention is the increase in sedentary behavior observed between the post-intervention and follow-up assessments in the control group, particularly in the leisure domain and in mentally-passive sedentary behavior, two indicators that, to some extent, are complementary. Although the precise causes of this increase cannot be determined, several hypotheses can be considered. One possibility is that this period coincided with contextual changes, such as the resumption of work and study routines, seasonal transitions, or periods of reduced mobility, which may have promoted the accumulation of sedentary behaviors (Bailey, 2021; Garriga et al., 2021; Holmes et al., 2021; Pronk, 2021). Baseline data were collected at the beginning of the year (a period typically associated with vacations and summer in Brazil), which may encourage greater daily movement. In contrast, subsequent assessments coincided with the return to regular work and study routines (post-intervention), the end of the academic semester (follow-up), and the transition to colder seasons, all of which may have contributed to increased sedentary time.

The control group maintained their usual routine but received biweekly psychoeducational materials providing guidance on mental health, physical activity, sedentary behavior, and sleep. These contents may have acted as a low-intensity intervention, potentially diluting between-group differences. Besides that, they were also placed on a waiting list for future participation in the intervention. Evidence suggests that even under control conditions, a placebo effect may occur, which could help partially explain the results observed (Jones et al., 2021). Despite this support, the absence of in-person sessions and practical strategies may have limited the adoption of sustained behavioral changes. In contrast, although the intervention group did not show a significant reduction in sedentary behavior, they were able to maintain stable levels over time. This stability may reflect a protective effect of the intervention in the face of a potentially unfavorable context.

Regarding potential moderators, the results showed that sex moderated the intervention’s effects on three indicators of sedentary behavior: cell phone use, leisure-time sedentary behavior, and mentally passive sedentary behavior. In all cases, a similar pattern emerged, with increases among men and reductions among women over time. Although the literature suggests that women are generally more physically inactive than men (Nikitara et al., 2021), this finding indicates that they may be more receptive to health-focused intervention content. Overall, women tend to show greater interest in self-care and disease prevention (Pattyn et al., 2015; Schmader & Block, 2025), which may have positively influenced their attitudes toward sedentary behavior, particularly in settings where they have more autonomy over their time use, such as during leisure. Moreover, although this study did not directly assess the content accessed during sedentary periods, it is possible that women engaged with these technologies more intentionally, thereby reducing passive and prolonged sedentary behaviors. Such patterns suggest that interventions aiming to reduce sedentary behavior may benefit from sex-specific approaches, particularly when addressing discretionary time use.

Variations by age were evident in leisure-time sedentary behavior, with more pronounced reductions among middle-aged adults (37–59 years) compared to younger adults (20–36 years). While the former showed a decrease of up to 3.3 h per week between baseline and follow-up, the latter largely maintained their levels over time. This finding may be related to differences in how leisure time is used across age groups. Younger adults tend to engage more intensively and routinely with digital technologies, with their leisure time often revolving around social media, streaming services, and video games (Jeong & Nam, 2024; Sanders et al., 2024), behaviors that are key components of mentally passive sedentary behavior. In contrast, middle-aged adults may allocate their free time more diversely, incorporating offline activities or socially engaging pursuits, such as spending time with family and friends (Monma et al., 2016; Solhi et al., 2022). This age group may also have been more receptive to the intervention content, possibly due to greater awareness of the long-term health consequences of harmful habits like excessive sedentary behavior, particularly in the context of mental and physical health. Tailoring strategies to different age groups, especially in leisure contexts, may therefore increase the effectiveness of interventions targeting reductions in sedentary time.

Differences were also observed according to marital status. Sedentary time measured by accelerometry increased among participants with a partner, whereas those without a partner showed a reduction over time. This finding is particularly relevant because it was based on an objective measure, minimizing potential perception biases commonly associated with self-reported instruments. Unlike leisure-time sedentary behavior, which involves greater autonomy over time use and was assessed via questionnaire, total sedentary behavior captured by the accelerometer reflects all contexts of daily life. In this regard, participants with a partner may have maintained more structured habits due to greater stability in family and marital routines, which might also have been more sedentary (O’Donoghue et al., 2016). Conversely, those without a partner may have had more flexibility to reorganize their day and, perhaps, a greater need to leave the house, commute, or engage in social interactions, which could have contributed to the reduction in total sedentary time.

Categorizing sedentary behavior remains one of the main methodological challenges in the field, particularly in light of rapid changes in how people organize their daily routines. In the present study, we aimed to group sedentary behavior indicators based on both domains (e.g., leisure, work, and commuting) and cognitive aspects (mentally active or passive). However, it is important to recognize that the same behavior may carry different meanings depending on its context or the individual’s intention. For instance, the use of electronic devices such as computers, tablets, or smartphones may occur during either leisure or work, making it difficult to classify these behaviors as mentally active or passive. The use of multiple operationalizations and exploratory combinations in the present study was intended to capture this complexity, but it may also have contributed to variability in the results, particularly given the small sample size and the corresponding risk of Type I error. In addition, the instrument used (Mielke et al., 2020) was developed before the COVID-19 pandemic, a period that profoundly transformed patterns of work and leisure through remote and hybrid arrangements, potentially affecting the accuracy of domain-based classifications. Watching television, for example, was categorized as a leisure and mentally passive activity, although in some cases it may involve substantial attention and cognitive engagement. Similarly, passive transportation was classified as mentally passive, even though activities like driving require continuous focus. These considerations highlight the need for caution when interpreting the data, especially in studies exploring the relationship between sedentary behavior and mental health. In some circumstances, mentally active sedentary behaviors (such as reading, studying, or performing professional tasks), as well as those occurring in occupational contexts, may even serve a protective role against depressive symptoms (Hallgren et al., 2018, 2020). This underscores the importance of adopting more nuanced approaches in future research on this topic.

This study has several limitations that should be acknowledged. Because the original sample size was calculated for the primary outcome (depressive symptoms), the study was underpowered for detecting modest changes in sedentary behavior. Therefore, the findings for sedentary behavior should be interpreted with caution. The generalizability of the findings is also limited by the characteristics of the sample. The study was conducted in a single urban area in southern Brazil, a country marked by wide regional, socioeconomic, and cultural diversity, which may restrict the applicability of the results to other contexts. In addition, the sample was predominantly composed of women, which reduces external validity to the male population. Nevertheless, this composition reflects well-established patterns in the literature, such as the higher prevalence of depressive symptoms among women compared to men (Schuch et al., 2017) and women’s greater participation in community-based health and behavioral programs (Deeks et al., 2009; Gorini et al., 2025). These factors may also help explain the higher engagement and retention rates observed in this study. An additional consideration relates to participant eligibility. The PHQ-9 score ≥ 9 was used as an inclusion criterion to identify individuals with elevated depressive symptoms. Under this approach, participants could meet the inclusion threshold based on higher endorsement of somatic or non-specific symptoms (e.g., sleep disturbance, appetite change, or concentration difficulties), without necessarily reporting depressive symptoms required for a formal diagnosis according to established diagnostic guidelines, such as anhedonia or depressed mood. As a result, the study population may include individuals experiencing elevated distress or subclinical symptom profiles rather than clinically diagnosed depressive disorders. Accordingly, the sample is more appropriately characterized as individuals at high risk for depression or presenting significant depressive symptomatology, which should be taken into account when interpreting the clinical framing of the findings and their generalizability to populations with confirmed depressive disorders. Access to an electronic device was also required to ensure feasibility of remote sessions and digital communication. Importantly, no individuals were excluded for this reason, and recent national data indicate high levels of mobile phone ownership across socioeconomic strata in Brazil (Brazilian Institute of Geography and Statistics, 2024). Even so, in other implementation contexts, this criterion may disproportionately affect groups with limited digital access. Researchers adopting similar intervention models should remain attentive to this potential constraint, even though it did not introduce socioeconomic bias in the present study.

Sedentary behavior assessment also presents important limitations. Although accelerometry provides an objective measure, the cutoff used to classify sedentary time was derived from previous calibration studies with wrist-worn devices in adult populations (Hildebrand et al., 2014). Despite its broad adoption in the literature, there is still no universal consensus on optimal thresholds for wrist placement, particularly among individuals with depressive symptoms, which may have led to minor misclassification. In addition, self-reported sedentary behavior is subject to recall and interpretation bias. The instrument used to assess self-reported sedentary behavior included separate questions for weekdays and Sundays. For analytical purposes, only weekday data were considered, as Sunday responses, without corresponding data for Saturdays, could distort the results by failing to represent typical weekly behavioral patterns. This decision also aimed to enhance comparability across indicators and reduce the risk of generating multiple combinations and interpretations without substantial improvements in precision. However, this approach may also have slightly reduced the sensitivity to detect variations in sedentary behavior that occur during weekends, which could have influenced the magnitude of observed effects. In individuals with depressive symptoms, sedentary behavior during weekends may also be qualitatively different, often being more leisure-oriented, socially embedded, or mood-related, rather than structured by work or routine demands. Excluding weekend data may therefore have limited the ability to capture context-specific changes in discretionary sedentary behavior, potentially reducing sensitivity to intervention-related effects. Additionally, it was not possible to gather information about the content accessed on electronic devices, which limits the ability to assess the cognitive quality of sedentary behaviors. This gap may affect the interpretation of certain domains, such as leisure time and mobile phone use, particularly in the current context of blurred boundaries between work and personal activities. Together, these methodological considerations may also have introduced measurement variability and should be taken into account when interpreting the findings.

Despite these limitations, this study has several important strengths. The combined use of subjective and objective measures of sedentary behavior provides a more nuanced understanding of this behavior across different contexts. The longitudinal design, which included both short- and long-term assessments, enabled the identification of changes over time in response to the intervention. Additionally, the analysis of potential moderators highlighted subgroup-specific patterns of response and reinforces the importance of considering sociodemographic and other individual factors in the design and implementation of health interventions. Together, these elements contribute to advancing knowledge on the effects of interventions targeting sedentary behavior in the context of mental health.

## Conclusion

The results indicate that the intervention was not effective in reducing sedentary behavior in either the short or long term among adults with moderate to severe depressive symptoms. Nevertheless, the identification of moderated effects by sex, age, and marital status underscores the importance of considering sociodemographic characteristics in the design and evaluation of behavior change strategies. Future interventions should adopt more integrated approaches, incorporating specific content on sedentary behavior and its various manifestations, while also acknowledging its potential interactions with physical activity and sleep. Moreover, the use of more refined methods, capable of capturing not only the duration but also the context and nature of sedentary activities, may enhance our understanding of their impact on mental health. In clinical and community settings, such knowledge can inform the development of more personalized interventions, with greater potential for long-term impact and sustainability.

## Electronic Supplementary Material

Below is the link to the electronic supplementary material.

Supplementary Material 1

Supplementary Material 2

## Data Availability

Available upon author’s request.
