# Supplementary material for: Moderators and short and long-term effects of a behavioral intervention on sedentary behavior among adults with depressive symptoms: results from a randomized clinical trial
Source: J Behav Med. 2026 Apr 17;49(4):571–87. doi: 10.1007/s10865-026-00636-8 (PMC13385158; doi:10.1007/s10865-026-00636-8)
Supplement: Supplementary file 2 — Supplementary Material 2 [file 10865_2026_636_MOESM2_ESM.docx]

*Table 1.* Characteristics of *Vincular* Project participants (n = 39 CG; n = 39 IG) analyzed at baseline.

| **Variables** | **Control group** | **Intervention group** | **p-value** |
| --- | --- | --- | --- |
|  | **x̅ (± sd)** | **x̅ (± sd)** |  |
| Age (years) | 37.4 (±10.29) | 37.5 (±10.26) | 0.939 |
| Educational level (years) | 16.2 (±3.93) | 15.7 (±4.90) | 0.648 |
| Depressive symptoms (PHQ-9 score) | 17.5 (±4.04) | 17.3 (±4.72) | 0.837 |
| Sleep by accelerometer (minutes/day) | 378.3 (±68.05) | 356.2 (±59.14) | 0.130 |
| Light PA by accelerometer (minutes/day) | 159.3 (±36.79) | 162.3 (±39.71) | 0.734 |
| Moderate PA by accelerometer (minutes/day) | 93.2 (±42.71) | 85.4 (±29.36) | 0.353 |
| Vigorous PA by accelerometer (minutes/day) | 3.0 (±3.29) | 13.1 (±47.63) | 0.191 |
| Walking by SIMPAQ (hours/day) | 0.6 (±0.81) | 0.9 (±1.24) | 0.274 |
| Physical exercises by SIMPAQ (hours/day) | 0.3 (±0.43) | 0.5 (±0.53) | 0.090 |
| Other PA by SIMPAQ (hours/day) | 2.8 (±1.98) | 2.4 (±1.88) | 0.384 |
| Sleep duration by SIMPAQ (hours/day) | 8.8 (±1.96) | 8.6 (±2.14) | 0.540 |
| Sleep quality by questionnaire (PSQI score) | 9.8 (±2.42) | 10.6 (±2.47) | 0.344 |
|  | **n (%)** | **n (%)** |  |
| Sex |  |  | 1.000 |
| Male | 9 (23.1) | 9 (23.1) |  |
| Female | 30 (76.9) | 30 (76.9) |  |
| Skin color |  |  | 0.620 |
| Withe | 28 (73.7) | 26 (66.7) |  |
| Other | 10 (26.3) | 13 (33.3) |  |
| Marital status |  |  | 0.802 |
| With a partner | 11 (28.9) | 10 (25.6) |  |
| Without a partner | 27 (71.1) | 29 (74.4) |  |
| Diagnosis of mental illness |  |  | 0.479 |
| No | 12 (30.8) | 16 (41.0) |  |
| Yes | 27 (69.2) | 23 (59.0) |  |
| Depression diagnosis |  |  | 1.000 |
| No | 21 (53.8) | 21 (53.8) |  |
| Yes | 18 (46.2) | 18 (46.2) |  |
| Use of medication for depression | |  | 0.810 |
| No | 12 (30.8) | 14 (35.9) |  |
| Yes | 27 (69.2) | 25 (64.1) |  |

Notes: PHQ-9 = Patient Health Questionnaire-9 (range from 0 to 27 points); PA = physical activity; SIMPAQ = Simple Physical Activity Questionnaire; PSQI = Pittsburgh Sleep Quality Index (range from 0 to 21 points); x̅ = mean; ± sd = standard deviation; n = absolute frequency; % = relative frequency; p-values were obtained using the t test and chi-squared test.

*Table 2.* Sensitivity analysis (intention-to-treat) considering alternative categorizations of sedentary behavior: short and long-term effects and potential moderators of a behavioral intervention in adults with depressive symptoms (n = 78).

| **Indicators** | **Baseline** |  | **Post-intervention** |  | **Follow-up** |  | **ɳ2p** | | | **p-value** | | | | | |
| --- | --- | --- | --- | --- | --- | --- | --- | --- | --- | --- | --- | --- | --- | --- | --- |
|  | **x̅ (±se)** |  | **x̅ (±se)** |  | **x̅ (±se)** |  | **g** | **t** | **g*t** | **g** | **t** | **g*t** | **g*t*sex** | **g*t*age** | **g*t*marital_status** |
| **Leisure (TV + cell phone)** |  |  |  |  |  |  | 0.002 | 0.089 | 0.022 | 0.685 | **<0.001** | 0.159 | **0.076** | 0.124 | 0.668 |
| Control group | 5.6 (±0.62) | * | 4.3 (±0.39) | * | 5.0 (±0.35) | * |  |  |  |  |  |  |  |  |  |
| Intervention group | 6.6 (±0.91) |  | 4.8 (±0.53) |  | 4.4 (±0.37) |  |  |  |  |  |  |  |  |  |  |
| **Occupational (sitting time^a^ + computer)** |  |  |  |  |  |  | 0.001 | 0.020 | 0.002 | 0.879 | 0.280 | 0.834 | 0.119 | 0.652 | 0.723 |
| Control group | 8.9 (±1.00) |  | 9.4 (±0.72) |  | 9.8 (±0.74) |  |  |  |  |  |  |  |  |  |  |
| Intervention group | 8.7 (±0.88) |  | 9.6 (±0.84) |  | 9.4 (±0.76) |  |  |  |  |  |  |  |  |  |  |
| **Mentally-passive (TV + commuting)** |  |  |  |  |  |  | 0.003 | 0.058 | 0.011 | 0.605 | **<0.001** | 0.255 | 0.170 | 0.382 | 0.505 |
| Control group | 3.4 (±0.40) | * | 2.2 (±0.23) | * % | 3.0 (±0.21) | % |  |  |  |  |  |  |  |  |  |
| Intervention group | 2.9 (±0.53) |  | 2.4 (±0.27) |  | 2.7 (±0.29) |  |  |  |  |  |  |  |  |  |  |
| **Mentally-active (sitting time^a^ + cell phone + computer)** |  |  |  |  |  |  | 0.001 | 0.001 | 0.015 | 0.762 | 0.987 | 0.140 | **<0.001** | 0.547 | 0.358 |
| Control group | 12.2 (±1.06) |  | 12.3 (±0.74) |  | 13.0 (±0.74) |  |  |  |  |  |  |  |  |  |  |
| Intervention group | 13.2 (±0.99) |  | 13.0 (±0.87) |  | 12.4 (±0.83) |  |  |  |  |  |  |  |  |  |  |
| **Mentally-passive (TV + commuting + cell phone)** |  |  |  |  |  |  | 0.003 | 0.071 | 0.014 | 0.653 | **0.001** | 0.142 | **0.039** | 0.322 | 0.884 |
| Control group | 6.7 (±0.73) | * | 5.1 (±0.41) | * | 6.2 (±0.36) |  |  |  |  |  |  |  |  |  |  |
| Intervention group | 7.4 (±0.91) |  | 5.8 (±0.54) |  | 5.6 (±0.39) |  |  |  |  |  |  |  |  |  |  |
| **Mentally-active (sitting time^a^ + computer)** |  |  |  |  |  |  | 0.001 | 0.020 | 0.002 | 0.879 | 0.280 | 0.834 | 0.119 | 0.652 | 0.723 |
| Control group | 8.9 (±1.00) |  | 9.4 (±0.72) |  | 9.8 (±0.74) |  |  |  |  |  |  |  |  |  |  |
| Intervention group | 8.7 (±0.88) |  | 9.6 (±0.84) |  | 9.4 (±0.76) |  |  |  |  |  |  |  |  |  |  |
| **Mentally-passive (TV + commuting + computer)** |  |  |  |  |  |  | 0.001 | 0.029 | 0.017 | 0.983 | **0.017** | 0.105 | 0.161 | 0.164 | 0.546 |
| Control group | 6.5 (±0.62) |  | 5.3 (±0.48) | % | 6.6 (±0.44) | % |  |  |  |  |  |  |  |  |  |
| Intervention group | 6.1 (±0.68) |  | 6.0 (±0.50) |  | 6.2 (±0.48) |  |  |  |  |  |  |  |  |  |  |
| **Mentally-active (sitting time^a^ + cell phone)** |  |  |  |  |  |  | 0.001 | 0.005 | 0.016 | 0.841 | 0.710 | 0.322 | **0.006** | 0.833 | 0.410 |
| Control group | 9.1 (±0.70) |  | 9.2 (±0.47) |  | 9.4 (±0.52) |  |  |  |  |  |  |  |  |  |  |
| Intervention group | 9.9 (±0.79) |  | 9.4 (±0.61) |  | 8.9 (±0.58) |  |  |  |  |  |  |  |  |  |  |

Notes: a = sitting time at work and school, technical course, college or other course; x̅ = mean; ± se = standard error; g = difference between groups; t = difference between times; g*t = interaction between group and time; g*t*sex = interaction between group, time, and sex; g*t*age = interaction between group, time, and age; g*t*marital_status = interaction between group, time, and marital status; η2p = partial eta squared; * = difference to baseline; % = difference to post-intervention.

*Table 3.* Sensitivity analysis (intention-to-treat) considering alternative categorizations of sedentary behavior: short and long-term effects of a behavioral intervention on different indicators of sedentary behavior, according to sex (n = 78).

| **Indicators** | **Men** | | | |  | **Women** | | | |
| --- | --- | --- | --- | --- | --- | --- | --- | --- | --- |
|  | **Baseline** | **Post-intervention** | **Follow-up** | **diff-in-diff (a / b)** |  | **Baseline** | **Post-intervention** | **Follow-up** | **diff-in-diff (a / b)** |
|  | **x̅ (±se)** | **x̅ (±se)** | **x̅ (±se)** |  |  | **x̅ (±se)** | **x̅ (±se)** | **x̅ (±se)** |  |
| **Leisure (TV + cell phone)** |  |  |  | 2.6 / 0.5 |  |  |  |  | -1.3 / -2.2 |
| Control group | 6.6 (±1.59) | 4.2 (±1.16) | 5.5 (±0.52) |  |  | 5.4 (±0.63) | 4.4 (±0.37) | 4.9 (±0.43) |  |
| Intervention group | 4.8 (±1.18) | 5.0 (±1.14) | 4.2 (±1.03) |  |  | 7.1 (±1.11) | 4.8 (±0.60) | 4.4 (±0.37) |  |
| **Mentally-active (sitting time^a^ + cell phone + computer)** | |  |  | 4.7 / 3.0 |  |  |  |  | -2.0 / -3.1 |
| Control group | 16.1 (±1.56) | 13.3 (±1.72) | 12.9 (±1.93) |  |  | 10.9 (±1.20) | 12.0 (±0.80) | 13.0 (±0.77) |  |
| Intervention group | 12.1 (±1.53) | 14.0 (±1.58) | 11.9 (±1.54) |  |  | 13.5 (±1.19) | 12.6 (±1.02) | 12.5 (±0.98) |  |
| **Mentally-passive (TV + commuting + cell phone)** |  |  |  | 3.9 / 1.2 |  |  |  |  | -1.1 / -1.9 |
| Control group | 8.0 (±2.36) | 4.7 (±1.13) | 6.7 (±0.45) |  |  | 6.3 (±0.62) | 5.2 (±0.41) | 6.0 (±0.44) |  |
| Intervention group | 5.4 (±1.09) | 6.0 (±1.10) | 5.3 (±1.07) |  |  | 7.9 (±1.11) | 5.7 (±0.63) | 5.7 (±0.39) |  |
| **Mentally-active (sitting time^a^ + cell phone)** |  |  |  | 3.7 / 1.9 |  |  |  |  | -2.0 / -2.3 |
| Control group | 11.2 (±1.11) | 9.3 (±1.17) | 9.0 (±1.44) |  |  | 8.5 (±0.82) | 9.2 (±0.49) | 9.5 (±0.53) |  |
| Intervention group | 8.7 (±1.23) | 10.5 (±0.98) | 8.4 (±1.03) |  |  | 10.3 (±0.94) | 9.0 (±0.72) | 9.0 (±0.69) |  |

Notes: a = sitting time at work and school, technical course, college or other course; x̅ = mean; ± se = standard error; diff-in-diff a = [(post-intervention value from IG – baseline value from IG) – (post-intervention value from CG – baseline value from CG)]; diff-in-diff b = [(follow-up value from IG – baseline value from IG) – (follow-up value from CG – baseline value from CG)].

*Table 4.* Per-protocol analysis of short and long-term effects and moderators of a behavioral intervention on different indicators of sedentary behavior in adults with depressive symptoms (accelerometry data: n = 34; CG: n = 19; IG: n = 15; SIMPAQ: n = 36; CG: n = 20; IG: n = 16; Mielke's Questionnaire: n = 38; CG: n = 22; IG: n = 16).

| **Indicators** | **Baseline** |  | **Post-intervention** |  | **Follow-up** |  | **ɳ2p** | | | **p-value** | | | | | |
| --- | --- | --- | --- | --- | --- | --- | --- | --- | --- | --- | --- | --- | --- | --- | --- |
|  | **x̅ (±se)** |  | **x̅ (±se)** |  | **x̅ (±se)** |  | **g** | **t** | **g*t** | **g** | **t** | **g*t** | **g*t*sex** | **g*t*age** | **g*t*marital_status** |
| **SB by accelerometer (minutes/day)** |  |  |  |  |  |  | 0.066 | 0.048 | 0.006 | 0.124 | 0.228 | 0.761 | 0.804 | 0.841 | 0.836 |
| Control group | 703.2 (±21.33) |  | 711.3 (±15.76) |  | 726.6 (±19.71) |  |  |  |  |  |  |  |  |  |  |
| Intervention group | 737.9 (±24.38) |  | 760.9 (±23.31) |  | 762.1 (±22.38) |  |  |  |  |  |  |  |  |  |  |
| **SB by SIMPAQ (hours/day)** |  |  |  |  |  |  | 0.001 | 0.002 | 0.031 | 0.955 | 0.937 | 0.295 | 0.172 | **0.092** | 0.293 |
| Control group | 9.2 (±0.71) |  | 9.6 (±0.66) |  | 10.1 (±0.59) |  |  |  |  |  |  |  |  |  |  |
| Intervention group | 9.9 (±0.83) |  | 9.8 (±0.66) |  | 9.1 (±0.79) |  |  |  |  |  |  |  |  |  |  |
| **TV time (hours/day)** |  |  |  |  |  |  | 0.004 | 0.066 | 0.012 | 0.697 | 0.064 | 0.621 | 0.580 | **0.054** | 0.796 |
| Control group | 1.4 (±0.33) |  | 0.9 (±0.20) |  | 1.5 (±0.29) |  |  |  |  |  |  |  |  |  |  |
| Intervention group | 1.6 (±0.41) |  | 1.3 (±0.35) |  | 1.5 (±0.50) |  |  |  |  |  |  |  |  |  |  |
| **Cell phone use (hours/day)** |  |  |  |  |  |  | 0.001 | 0.047 | 0.005 | 0.986 | 0.199 | 0.789 | **0.068** | 0.511 | 0.849 |
| Control group | 3.3 (±0.64) |  | 2.5 (±0.34) |  | 3.0 (±0.42) |  |  |  |  |  |  |  |  |  |  |
| Intervention group | 3.5 (±0.68) |  | 2.7 (±0.50) |  | 2.7 (±0.50) |  |  |  |  |  |  |  |  |  |  |
| **Computer at home (hours/day)** |  |  |  |  |  |  | 0.002 | 0.003 | 0.007 | 0.804 | 0.916 | 0.605 | 0.836 | 0.453 | 0.289 |
| Control group | 3.3 (±0.65) |  | 3.1 (±0.58) |  | 3.2 (±0.60) |  |  |  |  |  |  |  |  |  |  |
| Intervention group | 3.4 (±0.82) |  | 3.6 (±0.79) |  | 3.3 (±0.79) |  |  |  |  |  |  |  |  |  |  |
| **Sitting time at work and school (hours/day)** |  |  |  |  |  |  | 0.001 | 0.038 | 0.015 | 0.849 | 0.280 | 0.608 | 0.330 | **0.006** | 0.958 |
| Control group | 5.6 (±0.80) |  | 5.9 (±0.65) |  | 5.4 (±0.75) |  |  |  |  |  |  |  |  |  |  |
| Intervention group | 5.2 (±0.72) |  | 6.3 (±0.94) |  | 5.8 (±0.89) |  |  |  |  |  |  |  |  |  |  |
| **Passive commuting (hours/day)** |  |  |  |  |  |  | 0.006 | 0.057 | 0.033 | 0.621 | **0.012** | 0.368 | 0.578 | 0.134 | **0.094** |
| Control group | 1.0 (±0.44) |  | 0.7 (±0.16) |  | 1.0 (±0.11) | % |  |  |  |  |  |  |  |  |  |
| Intervention group | 0.8 (±0.25) |  | 0.9 (±0.30) |  | 1.5 (±0.25) |  |  |  |  |  |  |  |  |  |  |
| **Leisure (TV + cell phone + computer)** |  |  |  |  |  |  | 0.003 | 0.088 | 0.022 | 0.726 | **0.021** | 0.121 | **0.070** | **0.074** | 0.490 |
| Control group | 8.1 (±0.90) |  | 6.4 (±0.75) | * | 7.7 (±0.80) |  |  |  |  |  |  |  |  |  |  |
| Intervention group | 8.5 (±0.99) |  | 7.5 (±1.00) |  | 7.4 (±0.95) |  |  |  |  |  |  |  |  |  |  |
| **Occupational (sitting time at work and school)** |  |  |  |  |  |  | 0.001 | 0.038 | 0.015 | 0.849 | 0.280 | 0.608 | 0.330 | **0.006** | 0.958 |
| Control group | 5.6 (±0.80) |  | 5.9 (±0.65) |  | 5.4 (±0.75) |  |  |  |  |  |  |  |  |  |  |
| Intervention group | 5.2 (±0.72) |  | 6.3 (±0.94) |  | 5.8 (±0.89) |  |  |  |  |  |  |  |  |  |  |
| **Mentally-passive (TV + commuting + cell phone + computer)** | |  |  |  |  |  | 0,006 | 0.079 | 0.016 | 0.617 | **0.001** | 0.186 | **0.089** | 0.321 | 0.999 |
| Control group | 9.1 (±1.08) |  | 7.1 (±0.72) |  | 8.7 (±0.78) | % |  |  |  |  |  |  |  |  |  |
| Intervention group | 9.3 (±0.84) |  | 8.4 (±1.02) |  | 8.9 (±0.95) |  |  |  |  |  |  |  |  |  |  |
| **Mentally-active (sitting time at work and school)** | |  |  |  |  |  | 0.001 | 0.038 | 0.015 | 0.849 | 0.280 | 0.608 | 0.330 | **0.006** | 0.958 |
| Control group | 5.6 (±0.80) |  | 5.9 (±0.65) |  | 5.4 (±0.75) |  |  |  |  |  |  |  |  |  |  |
| Intervention group | 5.2 (±0.72) |  | 6.3 (±0.94) |  | 5.8 (±0.89) |  |  |  |  |  |  |  |  |  |  |

Notes: SB = sedentary behavior; SIMPAQ = Simple Physical Activity Questionnaire; CG = control group; IG = intervention group; x̅ = mean; ± se = standard error; g = difference between groups; t = difference between times; g*t = interaction between group and time; g*t*sex = interaction between group, time, and sex; g*t*age = interaction between group, time, and age; g*t*marital_status = interaction between group, time, and marital status; η2p = partial eta squared; * = difference to baseline; % = difference to post-intervention.

*Table 5.* Per-protocol analysis of short and long-term effects of a behavioral intervention on different indicators of sedentary behavior according to sex, age, and marital status (accelerometry data: n = 34; CG: n = 19; IG: n = 15; SIMPAQ: n = 36; CG: n = 20; IG: n = 16; Mielke's Questionnaire: n = 38; CG: n = 22; IG: n = 16).

| **Indicators** | **Men** | | | |  | **Women** | | | |
| --- | --- | --- | --- | --- | --- | --- | --- | --- | --- |
|  | **Baseline** | **Post-intervention** | **Follow-up** | **diff-in-diff (a / b)** |  | **Baseline** | **Post-intervention** | **Follow-up** | **diff-in-diff (a / b)** |
|  | **x̅ (±se)** | **x̅ (±se)** | **x̅ (±se)** |  |  | **x̅ (±se)** | **x̅ (±se)** | **x̅ (±se)** |  |
| **Cell phone use** |  |  |  | 3.0 / 1.2 |  |  |  |  | -1.2 / -1.2 |
| Control group | 5.2 (±1.90) | 2.7 (±0.49) | 4.1 (±.75) |  |  | 2.6 (±0.38) | 2.4 (±0.43) | 2.6 (±0.46) |  |
| Intervention group | 2.3 (±0.48) | 2.8 (±1.18) | 2.4 (±0.46) |  |  | 4.0 (±0.93) | 2.6 (±0.49) | 2.8 (±0.70) |  |
| **Leisure (TV + cell phone + computer)** |  |  |  | 4.2 / 2.2 |  |  |  |  | -0.9 / -1.9 |
| Control group | 11.0 (±1.44) | 7.8 (±1.47) | 9.5 (±1.17) |  |  | 7.0 (±0.99) | 5.9 (±0.84) | 7.0 (±0.95) |  |
| Intervention group | 6.6 (±1.24) | 7.6 (±1.61) | 7.3 (±1.76) |  |  | 9.4 (±1.23) | 7.4 (±1.25) | 7.5 (±1.12) |  |
| **Mentally-passive (TV + commuting + cell phone + computer)** | |  |  | 5.5 / 3.6 |  |  |  |  | -0.7 / -1.5 |
| Control group | 12.9 (±2.46) | 8.3 (±1.36) | 10.6 (±1.22) |  |  | 7.6 (±0.94) | 6.7 (±0.83) | 8.0 (±0.91) |  |
| Intervention group | 7.3 (±0.81) | 8.2 (±1.14) | 8.6 (±1.58) |  |  | 10.1 (±1.06) | 8.5 (±1.38) | 9.0 (±1.19) |  |
|  | **20 to 36 years** | | | |  | **37 to 59 years** | | | |
| **SB by SIMPAQ (hours/day)** |  |  |  | 0 / 0 |  |  |  |  | -1.0 / -4.7 |
| Control group | 9.5 (±0.83) | 10.1 (±0.66) | 9.8 (±0.66) |  |  | 8.6 (±1.31) | 8.5 (±1.48) | 10.7 (±1.19) |  |
| Intervention group | 10.1 (±0.92) | 10.7 (±0.71) | 10.4 (±0.87) |  |  | 9.5 (±1.59) | 8.4 (±1.08) | 6.9 (±1.03) |  |
| **TV time (hours/day)** |  |  |  | 0.8 / 0.2 |  |  |  |  | -1.2 / -0.8 |
| Control group | 1.4 (±0.44) | 0.6 (±0.19) | 1.4 (±0.34) |  |  | 1.5 (±0.39) | 1.7 (±0.37) | 1.8 (±0.50) |  |
| Intervention group | 1.2 (±0.44) | 1.2 (±0.44) | 1.4 (±0.74) |  |  | 2.3 (±0.72) | 1.3 (±0.56) | 1.8 (±0.50) |  |
| **Sitting time at work and school (hours/day)** |  |  |  | 2.0 / 2.4 |  |  |  |  | -1.0 / -1.7 |
| Control group | 5.2 (±0.88) | 5.9 (±0.69) | 5.3 (±0.83) |  |  | 6.5 (±1.71) | 6.0 (±1.51) | 5.7 (±1.64) |  |
| Intervention group | 4.6 (±0.89) | 7.3 (±1.27) | 7.1 (±1.23) |  |  | 6.3 (±1.10) | 4.8 (±1.03) | 3.8 (±0.64) |  |
| **Leisure (TV + cell phone + computer)** |  |  |  | 0.4 / -1.7 |  |  |  |  | 0.3 / 0.7 |
| Control group | 8.3 (±1.15) | 6.2 (±0.87) | 7.8 (±0.96) |  |  | 7.4 (±1.18) | 7.1 (±1.46) | 7.5 (±1.40) |  |
| Intervention group | 10.5 (±1.08) | 8.8 (±1.21) | 8.3 (±1.32) |  |  | 5.3 (±0.93) | 5.3 (±1.29) | 6.1 (±1.03) |  |
| **Occupational (sitting time at work and school)** |  |  |  | 2.0 / 2.4 |  |  |  |  | -1.0 / -1.7 |
| Control group | 5.2 (±0.88) | 5.9 (±0.69) | 5.3 (±0.83) |  |  | 6.5 (±1.71) | 6.0 (±1.51) | 5.7 (±1.64) |  |
| Intervention group | 4.6 (±0.89) | 7.3 (±1.27) | 7.1 (±1.23) |  |  | 6.3 (±1.10) | 4.8 (±1.03) | 3.8 (±0.64) |  |
| **Mentally-active (sitting time at work and school)** |  |  |  | 2.0 / 2.4 |  |  |  |  | -1.0 / -1.7 |
| Control group | 5.2 (±0.88) | 5.9 (±0.69) | 5.3 (±0.83) |  |  | 6.5 (±1.71) | 6.0 (±1.51) | 5.7 (±1.64) |  |
| Intervention group | 4.6 (±0.89) | 7.3 (±1.27) | 7.1 (±1.23) |  |  | 6.3 (±1.10) | 4.8 (±1.03) | 3.8 (±0.64) |  |
|  | **With a partner** | | | |  | **Without a partner** | | | |
| **Passive commuting (hours/day)** |  |  |  | 0.1 / 0.9 |  |  |  |  | 0.5 / 1.0 |
| Control group | 0.7 (±0.22) | 0.6 (±0.15) | 1.0 (±0.17) |  |  | 1.1 (±0.57) | 0.7 (±0.20) | 1.0 (±0.13) |  |
| Intervention group | 0.9 (±0.50) | 1.3 (±0.54) | 1.1 (±0.38) |  |  | 0.7 (±0.29) | 0.8 (±0.35) | 1.6 (±0.31) |  |

Notes: SB = sedentary behavior; SIMPAQ = Simple Physical Activity Questionnaire; x̅ = mean; ± se = standard error; diff-in-diff a = [(post-intervention value from IG – baseline value from IG) – (post-intervention value from CG – baseline value from CG)]; diff-in-diff b = [(follow-up value from IG – baseline value from IG) – (follow-up value from CG – baseline value from CG)].

*Table 6.* Sensitivity analysis (per-protocol) considering alternative categorizations of sedentary behavior: short and long-term effects and potential moderators of a behavioral intervention in adults with depressive symptoms (accelerometry data: n = 34; CG: n = 19; IG: n = 15; SIMPAQ: n = 36; CG: n = 20; IG: n = 16; Mielke's Questionnaire: n = 38; CG: n = 22; IG: n = 16).

| **Indicators** | **Baseline** |  | **Post-intervention** |  | **Follow-up** |  | **ɳ2p** | | | **p-value** | | | | | |
| --- | --- | --- | --- | --- | --- | --- | --- | --- | --- | --- | --- | --- | --- | --- | --- |
|  | **x (±ep)** |  | **x (±ep)** |  | **x (±ep)** |  | **g** | **t** | **g*t** | **g** | **t** | **g*t** | **g*t*sex** | **g*t*age** | **g*t*marital_status** |
| **Leisure (TV + cell phone)** |  |  |  |  |  |  | 0.001 | 0.093 | 0.014 | 0.832 | **0.006** | 0.313 | **0.009** | 0.232 | 0.991 |
| Control group | 4.8 (±0.70) |  | 3.4 (±0.37) |  | 4.5 (±0.56) |  |  |  |  |  |  |  |  |  |  |
| Intervention group | 5.1 (±0.75) |  | 3.9 (±0.70) |  | 4.2 (±0.74) |  |  |  |  |  |  |  |  |  |  |
| **Occupational (sitting time^a^ + computer)** |  |  |  |  |  |  | 0.001 | 0.020 | 0.002 | 0.805 | 0.399 | 0.465 | 0.317 | 0.165 | 0.820 |
| Control group | 8.9 (±1.28) |  | 9.0 (±1.05) |  | 8.6 (±1.15) |  |  |  |  |  |  |  |  |  |  |
| Intervention group | 8.7 (±1.20) |  | 9.9 (±1.54) |  | 9.1 (±1.40) |  |  |  |  |  |  |  |  |  |  |
| **Mentally-passive (TV + commuting)** |  |  |  |  |  |  | 0.012 | 0.099 | 0.018 | 0.530 | **0.002** | 0.466 | 0.392 | **0.015** | 0.220 |
| Control group | 2.4 (±0.49) |  | 1.5 (±0.26) |  | 2.6 (±0.28) | % |  |  |  |  |  |  |  |  |  |
| Intervention group | 2.3 (±0.49) |  | 2.2 (±0.43) |  | 3.0 (±0.60) |  |  |  |  |  |  |  |  |  |  |
| **Mentally-active (sitting time^a^ + cell phone + computer)** |  |  |  |  |  |  | 0.002 | 0.013 | 0.019 | 0.808 | 0.584 | 0.502 | **0.001** | 0.752 | 0.691 |
| Control group | 12.2 (±1.12) |  | 11.5 (±1.07) |  | 11.5 (±1.15) |  |  |  |  |  |  |  |  |  |  |
| Intervention group | 12.1 (±1.22) |  | 12.6 (±1.54) |  | 11.8 (±1.49) |  |  |  |  |  |  |  |  |  |  |
| **Mentally-passive (TV + commuting + cell phone)** |  |  |  |  |  |  | 0.004 | 0.075 | 0.007 | 0.698 | **0.001** | 0.509 | **0.006** | 0.522 | 0.629 |
| Control group | 5.7 (±1.03) |  | 4.0 (±0.41) |  | 5.5 (±0.53) | % |  |  |  |  |  |  |  |  |  |
| Intervention group | 5.8 (±0.71) |  | 4.8 (±0.78) |  | 5.6 (±0.77) |  |  |  |  |  |  |  |  |  |  |
| **Mentally-active (sitting time^a^ + computer)** |  |  |  |  |  |  | 0.002 | 0.025 | 0.018 | 0.805 | 0.399 | 0.465 | 0.317 | 0.165 | 0.820 |
| Control group | 8.9 (±1.28) |  | 9.0 (±1.05) |  | 8.6 (±1.15) |  |  |  |  |  |  |  |  |  |  |
| Intervention group | 8.7 (±1.20) |  | 9.9 (±1.54) |  | 9.1 (±1.40) |  |  |  |  |  |  |  |  |  |  |
| **Mentally-passive (TV + commuting + computer)** |  |  |  |  |  |  | 0.009 | 0.062 | 0.028 | 0.552 | **0.036** | 0.258 | 0.224 | 0.375 | 0.834 |
| Control group | 5.8 (±0.74) |  | 4.6 (±0.63) |  | 5.7 (±0.70) | % |  |  |  |  |  |  |  |  |  |
| Intervention group | 5.8 (±0.64) |  | 5.7 (±0.78) |  | 6.2 (±0.86) |  |  |  |  |  |  |  |  |  |  |
| **Mentally-active (sitting time^a^ + cell phone)** |  |  |  |  |  |  | 0.001 | 0.008 | 0.009 | 0.859 | 0.767 | 0.710 | **0.008** | 0.354 | 0.791 |
| Control group | 8.9 (±0.67) |  | 8.4 (±0.65) |  | 8.4 (±0.79) |  |  |  |  |  |  |  |  |  |  |
| Intervention group | 8.7 (±0.73) |  | 9.0 (±0.93) |  | 8.5 (±1.06) |  |  |  |  |  |  |  |  |  |  |

Notes: CG = control group; IG = intervention group; a = sitting time at work and school, technical course, college or other course; x̅ = mean; ± se = standard error; g = difference between groups; t = difference between times; g*t = interaction between group and time; g*t*sex = interaction between group, time, and sex; g*t*age = interaction between group, time, and age; g*t*marital_status = interaction between group, time, and marital status; η2p = partial eta squared; % = difference to post-intervention.

*Table 7.* Sensitivity analysis (per-protocol) considering alternative categorizations of sedentary behavior: short and long-term effects of a behavioral intervention on different indicators of sedentary behavior, according to sex and age (accelerometry data: n = 34; CG: n = 19; IG: n = 15; SIMPAQ: n = 36; CG: n = 20; IG: n = 16; Mielke's Questionnaire: n = 38; CG: n = 22; IG: n = 16).

| **Indicators** | **Men** | | | |  | **Women** | | | |
| --- | --- | --- | --- | --- | --- | --- | --- | --- | --- |
|  | **Baseline** | **Post-intervention** | **Follow-up** | **diff-in-diff (a / b)** |  | **Baseline** | **Post-intervention** | **Follow-up** | **diff-in-diff (a / b)** |
|  | **x̅ (±se)** | **x̅ (±se)** | **x̅ (±se)** |  |  | **x̅ (±se)** | **x̅ (±se)** | **x̅ (±se)** |  |
| **Leisure (TV + cell phone)** |  |  |  | 3.7 / 1.5 |  |  |  |  | -1.3 / -1.7 |
| Control group | 5.8 (±1.78) | 3.1 (±0.61) | 5.6 (±0.75) |  |  | 4.3 (±0.67) | 3.5 (±0.45) | 4.1 (±0.69) |  |
| Intervention group | 3.2 (±1.10) | 4.2 (±1.86) | 4.5 (±1.74) |  |  | 5.9 (±0.86) | 3.8 (±0.55) | 4.0 (±0.71) |  |
| **Mentally-active (sitting time^a^ + cell phone + computer)** | |  |  | 5.8 / 4.5 |  |  |  |  | -0.8 / -1.3 |
| Control group | 16.2 (±1.99) | 13.3 (±2.56) | 12.0 (±2.77) |  |  | 10.7 (±1.15) | 10.8 (±1.06) | 11.4 (±1.18) |  |
| Intervention group | 9.7 (±1.51) | 12.6 (±2.48) | 10.0 (±2.04) |  |  | 13.2 (±1.52) | 12.5 (±1.94) | 12.6 (±1.90) |  |
| **Mentally-passive (TV + commuting + cell phone)** |  |  |  | 5.1 / 3.0 |  |  |  |  | -1.1 / -1.3 |
| Control group | 7.8 (±3.20) | 3.6 (±0.70) | 6.7 (±0.62) |  |  | 5.0 (±0.65) | 4.2 (±0.49) | 5.1 (±0.66) |  |
| Intervention group | 3.9 (±1.01) | 4.8 (±1.72) | 5.8 (±1.80) |  |  | 6.7 (±0.80) | 4.8 (±0.81) | 5.5 (±0.77) |  |
| **Mentally-active (sitting time^a^ + cell phone)** |  |  |  | 5.3 / 3.8 |  |  |  |  | -1.1 / -1.1 |
| Control group | 11.0 (±1.43) | 8.6 (±1.53) | 8.1 (±2.00) |  |  | 8.1 (±0.64) | 8.3 (±0.68) | 8.5 (±0.79) |  |
| Intervention group | 6.3 (±0.84) | 9.2 (±1.48) | 7.2 (±1.60) |  |  | 9.8 (±0.80) | 8.9 (±1.18) | 9.1 (±1.32) |  |
|  | **20 to 36 years** | | | |  | **37 to 59 years** | | | |
| **Mentally-passive (TV + commuting)** |  |  |  | 1.5 / 1.3 |  |  |  |  | -1.0 / -1.0 |
| Control group | 2.5 (±0.66) | 1.2 (±0.26) | 2.5 (±0.34) |  |  | 2.3 (±0.33) | 2.5 (±0.49) | 2.8 (±0.47) |  |
| Intervention group | 1.3 (±0.44) | 1.5 (±0.52) | 2.6 (±0.75) |  |  | 4.1 (±0.61) | 3.3 (±0.51) | 3.6 (±0.75) |  |

Notes: CG = control group; IG = intervention group; a = sitting time at work and school, technical course, college or other course; x̅ = mean; ± se = standard error; diff-in-diff a = [(post-intervention value from IG – baseline value from IG) – (post-intervention value from CG – baseline value from CG)]; diff-in-diff b = [(follow-up value from IG – baseline value from IG) – (follow-up value from CG – baseline value from CG)].
